# Supplementary material for: Super Secondary Structures of Proteins with Post-Translational Modifications in Colon Cancer
Source: Molecules. 2020 Jul 9;25(14):3144. doi: 10.3390/molecules25143144 (PMC7397127; doi:10.3390/molecules25143144)

Table S1. BLOCK 1 Rules for recognition and selection of a helical pair for a peptide fragment

1. Analysis of the secondary structure of the amino acid chain is carried out using the Dictionary for Secondary Structure of Protein. Search for sections of helixes of the following types: α-, 310-, and π- helix.

2. A helical pair is a portion of the protein structure that is formed by two helixes adjacent to the polypeptide chain connected by an unstructured fragment (coil) of arbitrary length and configuration.

3. The following types of helical pairs are found in proteins: α-α-corner, α-α-hairpin, and V- and L-structures.

4. Each helix of a pair can be inscribed in a cylinder. The cylinder axis is determined by the least squares method with the minimum deviation of the helix parameters from the ideal helix.

5. Two axes of the helixes determine the spatial structure of the helical pair. The spatial structure of the helical pair is determined by the interplanar distance (d) and the projections of the axes of the helixes on the plane.

6. The polygon area (S) of the intersection of the projections of the helixes depends on the relative position of the helixes (geometry) and the diameter of the cylinders of the helixes. The cylinder diameter is determined by the type of helix and the average size of the side chain.

Distinctive properties of types of helical pairs

1. α-α corner

- dense orthogonal packing of helixes;

- the projections of the helixes and the axis of the helixes intersect;

- the values of the interplanar distance and the minimum distance are equal ($d = r$);

- the intercoil distance within $d \approx$ (9-11) Å;

- the area and perimeter of the intersection of the projections of the helixes are larger than the other two- helix motifs;

- torsion θ and flat φ inter helix angles have values of 70-110°;

- the length of the waist (Np) is usually 4-5 amino acids.

2. α-α-hairpin

- dense antiparallel packing of helixes;

- the projections of the helixes intersect, but the axes of the helixes do not intersect;

- the range of the intercoil distance, d, is wider than that of the α-α corner;

- interhelix angles 0-30°;

- the length of the waist (Np) may vary;

- usually consists of two long helixes.

3. L-shaped structure

- dense bevelled packaging of helixes;

- the projections of the helixes and the axes of the helixes do not intersect (S ≈ 0 Å2, P ≈ 0 Å);

- interhelix angle has a value φ ≈ 90°;

- The waist length (Np) may vary.

4. V-shaped structure

- dense bevelled packaging of helixes;

- the projections of the helixes (most often) and the axes of the helixes do not intersect;

- area, S, and perimeter have values close to zero;

- interhelix angle φ ≈ 50°;

- coil (Np), as a rule, consists of one amino acid;

- length of the helixes, as a rule, does not exceed three to four turns.

Table S2. Data of colon cancer patients

N	Sex	Age, years	Diagnosis	Stage	Metastases
2	Female	56	colorectal cancer	IV	in the liver
3	Female	59	colorectal cancer	IIIc	No
4	Female	28	colorectal cancer	IIa	No
5	Female	69	colorectal cancer	IV	in the ovaries
6	Female	54	colorectal cancer	IV	in the liver
8	Male	85	colorectal cancer	IV	in the liver
9	Male	63	colorectal cancer	IV	in the lymph nodes
10	Female	43	colorectal cancer	I	No
11	Female	76	colorectal cancer	IIa	No
12	Male	46	colorectal cancer	IIa	No
13	Female	64	colorectal cancer	I	No
14	Female	41	colorectal cancer	IVb	in the liver
15	Female	69	colorectal cancer	IIIa	No
16	Female	81	colorectal cancer	I	No
18	Female	32	colorectal cancer	IIIa	No
19	Male	50	colorectal cancer	I	No
20	Female	60	colorectal cancer	I	No
21	Female	31	colorectal cancer	IIa	No
22	Female	55	anal canal	I	No
23	Female	64	colorectal cancer	IIIc	No
24	Male	45	colorectal cancer	IV	in the lymph nodes
25	Male	58	colorectal cancer	IIa	No
26	Female	60	colorectal cancer	IIa	No
28	Male	93	colorectal cancer	IIa	No
30	Female	70	colorectal cancer	IIa	No
31	Female	64	colorectal cancer	IIIb	No
42	Male	81	colorectal cancer	I	No
54	Male	58	colorectal cancer	IIIb	No

Data of healthy volunteers

Sample	Sex	Age, years
1	Female	53
2	Female	38
3	Female	58
4	Female	49
5	Male	70
6	Female	41
7	Male	61

8	Female	45
9	Male	46
10	Male	41
11	Female	55
12	Female	60
13	Female	56
14	Female	52
15	Male	59
16	Male	62
17	Male	39
18	Female	40
20	Female	39
22	Male	44
24	Female	46
25	Male	55
26	Female	42
28	Male	40
29	Male	44
30	Male	40
31	Male	44
32	Male	40
35	Female	48
37	Female	39
38	Male	39
42	Male	42
43	Male	39
44	Male	34
46	Male	53
47	Female	53
50	Female	47
51	Female	52
60	Male	64
61	Female	49
62	Female	45

The recorded raw data were converted to peak lists for search engines and deposited to the ProteomeXchange Consortium via the PRIDE partner repository with the dataset identifier PXD015163.

Table S3

UniProt AC	Protein name	Peptide*	ID PDB	Localization**	PTM	Number of samples	Stage***
		LKCASLQK	1AO6	199	ac (K)	11	I;II;IV<br>II-IV
ALBU	Serum albumin	HCIAEVENDEMPADLPSLAADFVESKDVC	1AO6	313	ac (K)	5	II-IV
		ADLAKYICENQDSISSK	1AO6	262	ac (K)	2	IV
		LVNEVTEFAKTCVADESAENCDK	1AO6	51	ac (K)	3	I; II; IV
		YICENQDSISSK	1AO6	263	p (Y)	2	II; IV
VTDB	Vitamin D-binding protein	VLEPTLKSLGECCDVEDSTTCFNAK	1J78	354	ac (K)	1	II
		SCESNSPFPVHPGTAECCTKEGLER	1J78	114	ac (K)	1	I
A2MG	Alpha-2-macroglobulin	SKAIGYLNTGYQR	4ACQ	1003	ac (K)	1	III
CO4A	Complement C4-A	LLATLCSAEVCQCAEGKCPR	5JPN	1594	ac (K)	1	I
XRCC6	X-ray repair cross-complementing protein 6	IISSDRDLLAVVFYGTEK	1JEQ	77	p (S)	1	IV
IC1	Plasma protease C1 inhibitor	LVLLNAIYLSAK	5DU3	272	p (Y)	2	I; IV

"Peptide*" indicates tryptic peptides
"Localization**" indicates locus aminoacid with PTM in the protein sequence (ID PDB)
"Stage***" indicates the stage of development of the cancer from I to IV

Figure S1 Three-dimensional protein structures extracted from the PDB DB with indicating spatial localizations of modified amino acids (highlighted in red and arrows in the figure). The insets show the corresponding proteins geometric of a supersecondary structure formed by two helices. (A) ALBU (PDB ID 1AO6) with 5 modified amino acids with lysine acetylation (Ac(K)) and tyrosine phosphosylation (P(Y)); (B) A2MG (PDB ID 4ACQ), (C) IC1 (PDB ID 5DU3), (D) VTDB (PDB ID 1J78), (E) CO4A (PDB ID 5JPN) with lysine acetylation; (F) XRCC-6 (PDB ID 1JEQ) with phosphorylated serine (P(S)).

(A) ALBU

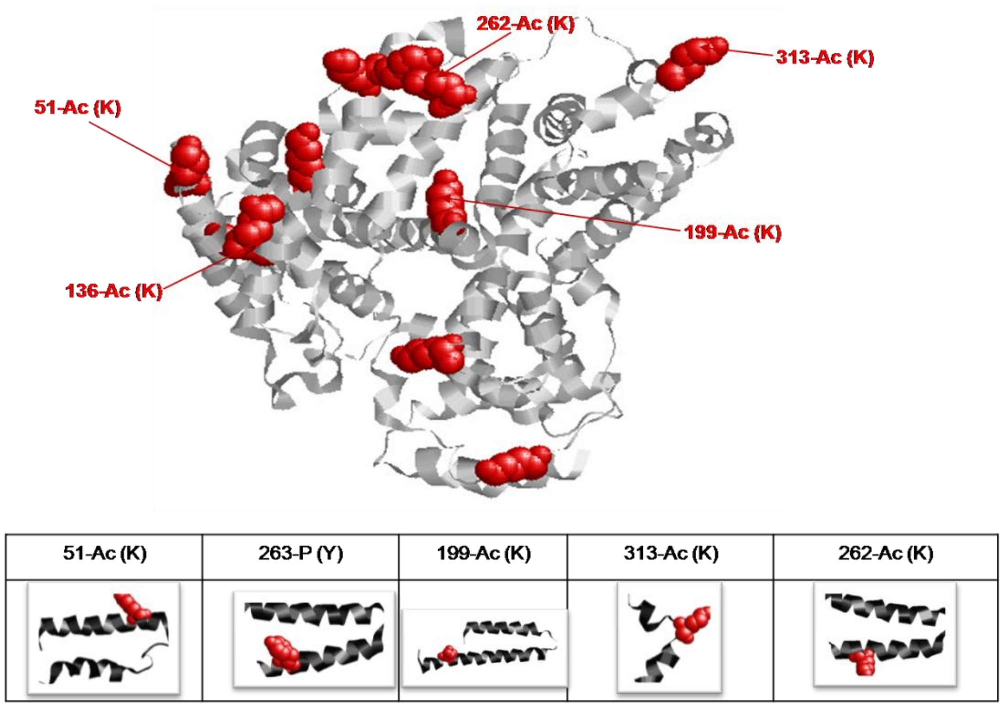

(B) A2MG

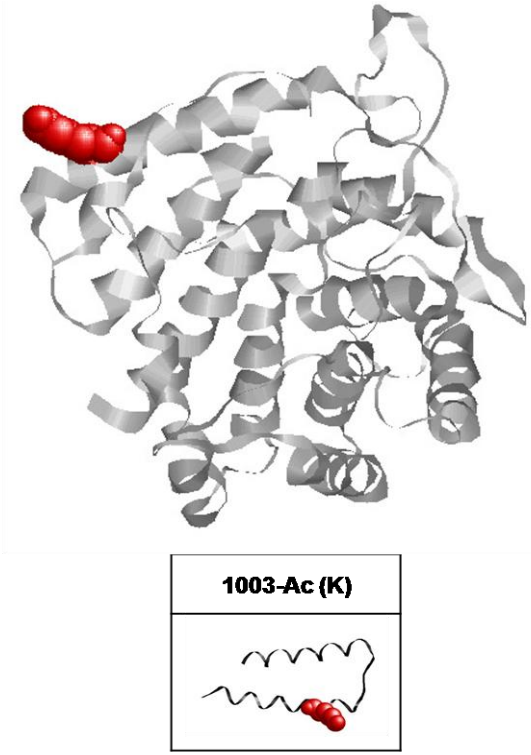

(C) IC1

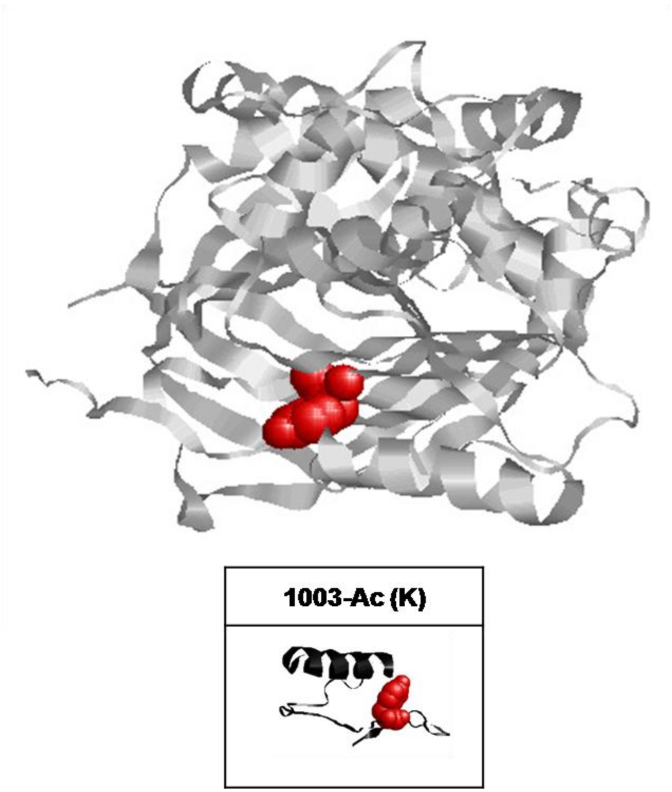

(D) VTDB

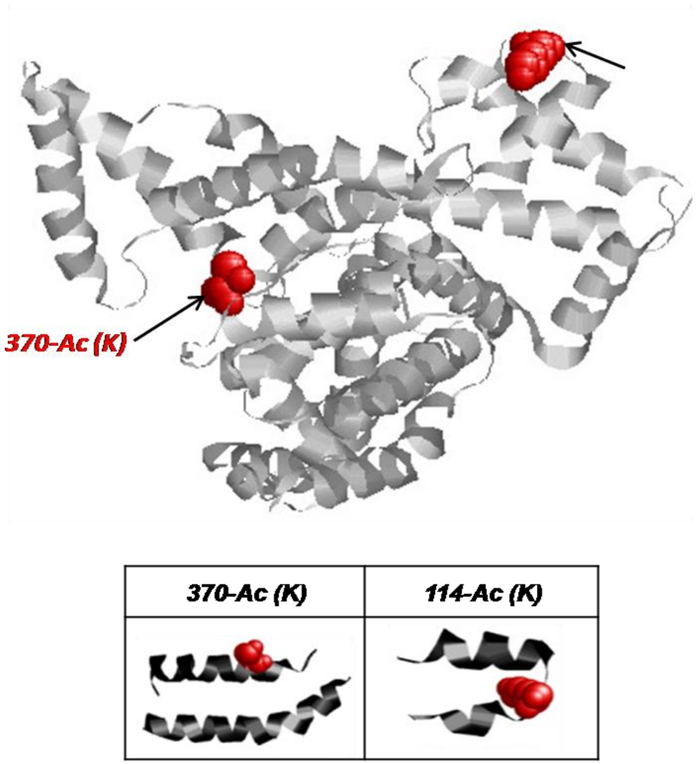

(E) CO4A

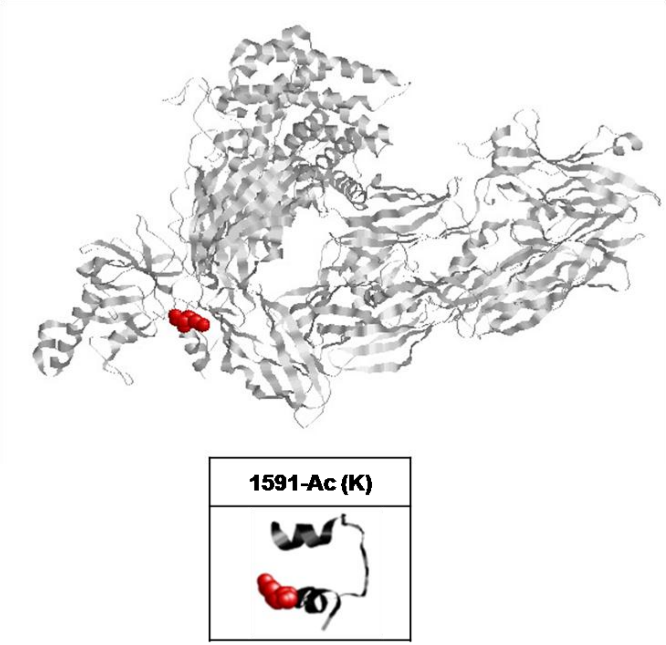

(F) XRCC-6

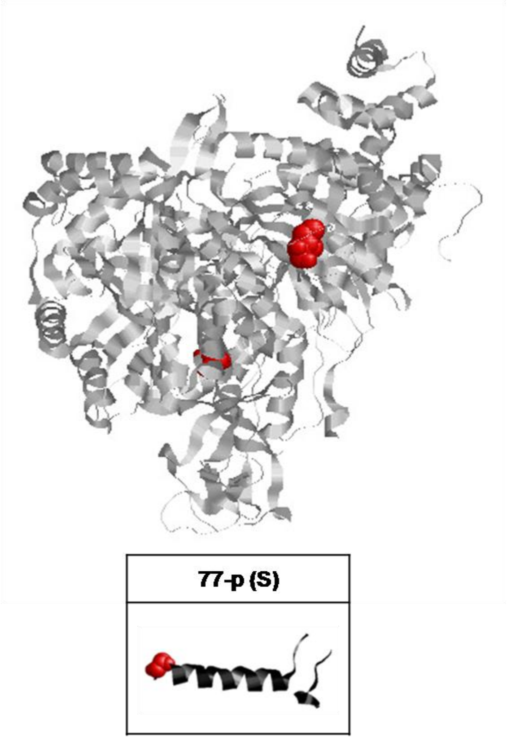

Supplement: Supplementary file 1 [file molecules-25-03144-s001.pdf]
